# Suppressor of cytokine signaling (SOCS) genes are downregulated in breast cancer

**DOI:** 10.1186/s12957-018-1529-9

**Published:** 2018-11-19

**Authors:** Soudeh Ghafouri-Fard, Vahid Kholghi Oskooei, Iman Azari, Mohammad Taheri

**Affiliations:** 1 Department of Medical Genetics, Shahid Beheshti University of Medical Sciences, P.O.Box: 19857-17443, Tehran, Iran; 2 Student Research Committee, Shahid Beheshti University of Medical Sciences, Tehran, Iran; 3 Urogenital Stem Cell Research Center, Shahid Beheshti University of Medical Sciences, Tehran, Iran

**Keywords:** Suppressor of cytokine signaling, Breast cancer, Expression

## Abstract

**Background:**

The suppressor of cytokine signaling (SOCS) family of proteins are inhibitors of the cytokine-activated Janus kinase/signal transducers and activators of transcription (JAK/STAT) signaling pathway. We aimed at evaluation of expression of SOCS genes in breast cancer.

**Methods:**

We evaluated expression of *SOCS1–3* and *SOCS5* genes in breast cancer samples compared with the corresponding adjacent non-cancerous tissues (ANCTs).

**Results:**

All assessed *SOCS* genes were significantly downregulated in tumoral tissues compared with ANCTs. *SOCS1* and *SOCS2* genes were significantly overexpressed in higher grade samples, but *SOCS3* had the opposite trend. Significant correlations were found between expression levels of *SOCS* genes. The *SOCS1* and *SOCS2* expression levels had the best specificity and sensitivity values respectively for breast cancer diagnosis.

**Conclusion:**

The current study provides further evidence for contribution of *SOCS* genes in breast cancer.

## Introduction

The suppressor of cytokine signaling (SOCS) family of proteins have been recognized as potent inhibitors of the cytokine-activated Janus kinase/signal transducers and activators of transcription (JAK/STAT) signaling pathway through which they also suppress cytokine signal transduction [1]. Apart from their role in the regulation of immune responses, tumor suppressor functions have been demonstrated for certain members of this family in various tissues. For instance, SOCS1 impedes proliferation signals relayed by several oncogenes in the hematopoietic lineage [2] and hepatic tissue [3]. Moreover, hypermethylation of *SOCS1* promoter has been shown in hepatocellular carcinoma [3], cervical cancer [4], and ovarian and breast cancer cells [5]. Such data implies that aberrant downregulation of *SOCS* genes might participate in the development of breast cancer as well. However, Evans et al. have shown upregulation of several members of SOCS family in MCF-7 and HCC1937, two cell lines that are regarded as prototypic breast cancer cell types. Moreover, they have demonstrated responsiveness of *SOCS1* and *SOCS3* promoters to regulation by cytokine or growth factor signals in spite of hypermethylation state of these promoters in these two cell lines [6]. Sutherland et al. have reported the inhibitory role of SOCS1 and SOCS2 but not SOCS3 on the growth of breast cancer cells and suggested hypermethylation of these genes as a mechanism for intensifying cytokine responsiveness and tumorigenesis process in breast tissue [5]. However, considering the difference in the expression of microenvironment-related genes in cancer cell lines and clinical samples, the data regarding expression pattern of *SOCS* genes in cell lines can be hardly adopted for clinical samples. The results of expression analysis of *SOCS* genes in clinical samples are inconsistent. Although Sasi et al. reported higher expression of *SOCS1* mRNA in breast tumor samples obtained from patients with earlier tumor stage and better survival [7], Raccurt et al. demonstrated constant higher expression of SOCS1–3 in tumor cells compared with normal adjacent epithelial and connective tissues [8]. Consistent with the results of the former study, expression of SOCS1 protein in breast cancer tissues has been associated with lower risk of identification of circulating tumor cells in the peripheral blood [9]. Based on the importance of SOCS-based strategies in treatment of cancer [10], assessment of expression of *SOCS* genes in clinical samples obtained from breast cancer patients is of practical value. Consequently, we designed the current study to evaluate the expression of *SOCS1–3* and *SOCS5* genes in invasive ductal carcinoma of the breast compared with the corresponding adjacent non-cancerous tissues (ANCTs).

## Material and methods

### Patients

Fifty-four patients with definite diagnosis of invasive ductal carcinoma of the breast participated in the study. The inclusion criteria were histopathological confirmation of invasive ductal carcinoma and availability of clinical data. Patients with other types of breast cancer and familial breast cancer and those who received prior chemo/radiotherapy were excluded from the study. The research protocol was approved by the ethical committee of Shahid Beheshti University of Medical Sciences. All methods were performed in accordance with the relevant guidelines and regulations. Informed written consent was obtained from all patients. Tumoral tissues and ANCTs were excised from all patients during surgery in Sina and Farmanieh hospitals. All tissue samples were transferred in liquid nitrogen to the genetic laboratory and stored in − 80 °C until gene expression experiments. Medical records of patients were assessed, and the relevant data was collected for correlation analysis.

### Expression analysis

Relative expressions of *SOCS* genes were assessed in tumoral tissues and ANCTs using quantitative real-time PCR technique. Briefly, total RNA was extracted from tissue samples using TRIzol™ Reagent (Invitrogen, Carlsbad, CA, USA), and cDNA was synthesized by using RevertAid First Strand cDNA Synthesis Kit (TaKaRa, Japan). TaqMan Fast Universal PCR Master Mix (Applied Biosystems, Foster City, USA) was used for expression analysis of genes. Expressions of genes were normalized to expression of *hypoxanthine-guanine phosphoribosyl transferase* (*HPRT*). The nucleotide sequences of primers are shown in Table 1.

**Table 1:** The nucleotide sequences of primers used for expression analysis

Gene name	Primer and probe sequence	Primer and probe length	Product length
*HPRT1*	F: AGCCTAAGATGAGAGTTC	18	88
	R: CACAGAACTAGAACATTGATA	21	
	FAM-CATCTGGAGTCCTATTGACATCGC-TAMRA	24	
*SOCS1*	F: TGGCCCCTTCTGTAGGATGG	20	109
	R: GGAGGAGGAAGAGGAGGAAGG	21	
	FAM-TGGCCCCTTCTGTAGGATGG-TAMRA	20	
*SOCS2*	F: ACGCGAACCCTTCTCTGACC	20	99
	R: CATTCCCGGAGGGCTCAAGG	20	
	FAM-CTCGGGCGGCCACCTGTCTTTGC-TAMRA	23	
*SOCS3*	F: GTGGAGAGGCTGAGGGACTC	20	111
	R: GGCTGACATTCCCAGTGCTC	20	
	FAM-CACCAAGCCAGCCCACAGCCAGG-TAMRA	23	
*SOCS5*	F: GTGACTCGGAAGAGGATACAACC	23	91
	R: CTAACATGGGTATGGCTGTCTCC	23	
	FAM-CGCTGCTTCTGCCTCCGTGACTGC-TAMRA	24	

All experiments were performed in duplicate in the rotor gene 6000 Corbett Real-Time PCR System.

### Estrogen receptor (ER)/progesterone receptor (PR)

ER/PR status was acquired from patients’ medical histories which were performed by immunohistochemical (IHC) staining. Staining of ≥ 5% of tumor cell nuclei was described as positive, while staining of lower percentages was reported as negative.

### HER2/neu

HER2/neu results were acquired from the medical reports of patient and were performed by IHC. Results of 0 to 2+ were regarded as negative and 3+ was considered as positive.

### Ki-67

Ki-67 status was assessed using IHC assays with the anti-human Ki-67 monoclonal antibody MIB1. The percentage of positively stained malignant cells among the total number of malignant cells was calculated. The results were reported as positive vs. negative.

### Statistical analysis

Student’s paired and unpaired *t* tests were used for analysis of differences in gene expression between paired and unpaired samples. The association between clinicopathological data and transcript levels of each gene was assessed using the chi-square test. Tukey’s honest significance test was used to find the difference between mean values of transcript levels between different groups. The expression fold change was measured using the efficiency corrected calculation models. The pairwise correlation between relative transcripts levels of genes was measured using the regression model. For all statistical tests, the level of significance was set at *P* < 0.05. The receiver operating characteristic (ROC) curve was plotted to evaluate the rightness of gene expression levels for differentiating tumoral vs. ANCTs. In order to evaluate gene expression probability cutoff, the Youden index (*j*) was used to maximize the difference between sensitivity (true-positive rate) and 1—specificity (false-positive rate). The precision of each marker for diagnosis of malignancy status was scored based on the area under curve (AUC) values using the following assumption: 0.90–1 = excellent (A), 0.80–0.90 = good (B), 0.70–0.80 = fair (C), 0.60–0.70 = poor (D), and 0.50–0.60 = fail (F).

## Results

### General demographic data of patients

General demographic data of patients are shown in Table 2.

**Table 2:** General demographic data of study participants

Variables	Values
Age (years) (mean ± SD)	51.79 ± 13.54 (29–81)
Menarche age (years) (mean ± SD)	13 ± 1.65 (10–18)
Menopause age (years) (mean ± SD)	44.91 ± 14.91 (38–60)
First pregnancy age (years) (mean ± SD)	18.04 ± 8.36 (14–32)
Breast feeding duration (months) (mean ± SD)	41.62 ± 34.1 (3–120)
Cancer stage (%)	
I	30.8
II	28.8
III	30.8
IV	9.6
Overall grade (%)	
I	17
II	49
III	34
Mitotic rate (%)	
I	45.2
II	42.9
III	11.9
Tumor size (%)	
< 2 cm	32
≥ 2 cm, < 5 cm	66
≥ 5 cm	2
Estrogen receptor (%)	
Positive	87.8
Negative	12.2
Progesterone receptor (%)	
Positive	77.1
Negative	22.9
Her2/neu expression (%)	
Positive	25
Negative	75
Ki67 expression (%)	
Positive	100
Negative	0

### Relative expression of *SOCS* in tumoral tissues vs. ANCTs

**Fig. 1:**
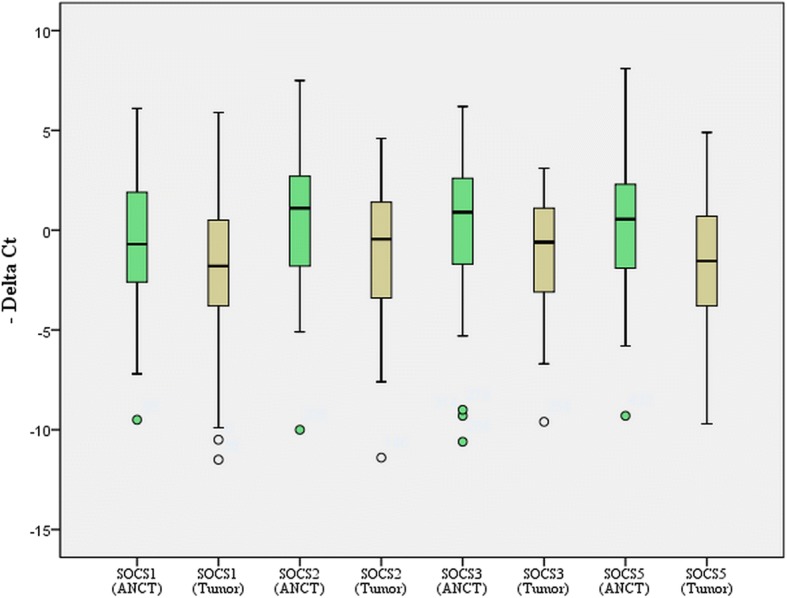
The relative expression of *SOCS* genes in tumoral tissues and ANCTs (*Y*-axis shows CT_reference gene_ − CT _target gene_)

All assessed *SOCS* genes were significantly downregulated when comparing total tumoral tissues with total ANCTs (*SOCS1*: expression ratio = 0.47, *P* = 0.033; *SOCS2*: expression ratio = 0.38, *P* = 0.008; *SOCS3*: expression ratio = 0.47, *P* = 0.027; and *SOCS5*: expression ratio = 0.35, *P* = 0.001). Figure 1 shows relative expression of *SOCS* genes in tumoral tissues and ANCTs.

### Association between relative expression of genes and patients’ clinicopathological data

We compared the expression level of each gene in each tumoral tissue vs. its corresponding ANCT and categorized patients based on these values to upregulation and downregulation groups. Next, we assessed associations between clinicopathological data and relative expressions of genes. No significant associations were found between the relative expression of genes in tumoral tissues and the ANCTs and patients’ clinicopathological data. Table 3 shows the results of association analysis between relative expressions of genes in tumoral tissues and ANCTs and patients’ clinicopathological data.

**Table 3:** The results of association analysis between relative expressions of genes in tumoral tissues compared with ANCTs and patients’ clinicopathological data (up/downregulation of genes was defined based on relative expression of each gene in tumoral tissue compared with the corresponding ANCT. Patients with higher expression of genes in tumoral tissue compared with the paired ANCT were classified as upregulation (fold change > 1) and vice versa)

	*SOCS1* upregulation	*SOCS1* downregulation	*P* value	*SOCS2* upregulation	*SOCS2* downregulation	*P* value	*SOCS3* upregulation	*SOCS3* downregulation	*P* value	*SOCS5* upregulation	*SOCS5* downregulation	*P* value
Age			0.35			0.5			0.84			0.68
< 55 years	11 (32.4%)	23 (67.6%)		9 (26.5%)	25 (73.5%)		11 (32.4%)	23 (67.6%)		10 (29.4%)	24 (70.6%)	
≥ 55 years	9 (45%)	11 (55%)		7 (35%)	13 (65%)		7 (35%)	13 (65%)		5 (25%)	15 (75%)	
Stage			0.95			0.81			0.8			0.86
1	5 (31.3%)	11 (68.7%)		5 (31.3%)	11 (68.7%)		4 (25%)	12 (75%)		3 (18.8%)	13 (81.2%)	
2	6 (40%)	9 (60%)		6 (40%)	9 (60%)		6 (40%)	9 (60%)		5 (33.3%)	10 (66.7%)	
3	7 (43.8%)	9 (56.3%)		4 (25%)	12 (75%)		5 (31.2%)	11 (68.8%)		5 (31.2%)	11 (68.8%)	
4	2 (40%)	3 (60%)		1 (20%)	4 (80%)		2 (40%)	3 (60%)		1 (20%)	4 (80%)	
Histological grade			0.32			0.18			0.08			0.19
1	2 (25%)	6 (75%)		1 (12.5%)	7 (87.5%)		0 (0%)	8 (100%)		0 (0%)	8 (100%)	
2	12 (52.2%)	11 (47.8%)		10 (43.5%)	13 (56.5%)		9 (39.1%)	14 (60.9%)		8 (34.8%)	15 (65.2%)	
3	5 (31.3%)	11 (68.8%)		3 (18.8%)	13 (81.2%)		7 (43.8%)	9 (56.2%)		4 (25%)	12 (75%)	
Mitotic rate			0.36			0.03			0.9			0.16
1	10 (52.6%)	9 (47.4%)		10 (52.6%)	9 (47.4%)		8 (42.1%)	11 (57.9%)		8 (42.1%)	11 (57.9%)	
2	6 (33.3%)	12 (66.7%)		4 (22.2%)	14 (77.8%)		6 (33.3%)	12 (66.7%)		4 (22.2%)	14 (77.8%)	
3	1 (20%)	4 (80%)		0 (0%)	5 (100%)		2 (40%)	3 (60%)		0 (0%)	5 (100%)	
Tumor size			0.26			0.29			1			0.38
< 2	4 (25%)	12 (75%)		5 (31.3%)	11 (68.8%)		5 (31.3%)	11 (68.8%)		4 (25%)	12 (75%)	
2–5	15 (45.5%)	18 (54.5%)		9 (27.3%)	24 (72.7%)		11 (33.3%)	12 (66.7%)		8 (24.2%)	25 (75.8%)	
> 5	0 (0%)	1 (100%)		1 (100%)	0 (0%)		0 (0%)	1 (100%)		1 (100%)	0 (0%)	
ER state			0.81			0.63			1			0.36
Positive	16 (38.1%)	26 (61.9%)		13 (31%)	29 (69%)		14 (33.3%)	28 (66.7%)		10 (23.8%)	32 (76.2%)	
Negative	3 (42.9%)	4 (57.1%)		2 (28.6%)	5 (71.4%)		2 (28.6%)	5 (71.4%)		3 (42.9%)	4 (57.1%)	
PR state			0.8			0.72			0.33			0.45
Positive	15 (40.5%)	22 (59.5%)		11 (29.7%)	26 (70.3%)		11 (29.7%)	26 (70.3%)		9 (24.3%)	28 (75%)	
Negative	4 (36.4%)	7 (63.6%)		4 (36.4%)	7 (63.6%)		5 (45.5%)	6 (54.5%)		4 (36.4%)	7 (63.6%)	
Her2 state			0.39			0.85			0.15			0.18
Positive	6 (50%)	6 (50%)		4 (33.3%)	8 (66.7%)		6 (50%)	6 (50%)		5 (41.7%)	7 (58.3%)	
Negative	13 (36.1%)	23 (63.9%)		11 (30.6%)	25 (69.4%)		10 (27.8%)	26 (72.2%)		8 (22.2%)	28 (77.8%)	
Breast feeding duration (months)			0.3			0.84			0.08			0.19
0	4 (50%)	4 (50%)		2 (25%)	6 (75%)		5 (62.5%)	3 (37.5%)		4 (50%)	4 (50%)	
1–30	8 (53.3%)	7 (46.7%)		6 (40%)	9 (60%)		6 (40%)	9 (60%)		6 (40%)	9 (60%)	
31–60	5 (27.8%)	13 (72.2%)		5 (27.8%)	13 (72.2%)		6 (33.3%)	12 (66.7%)		3 (16.7%)	15 (83.3%)	
61–120	3 (25%)	9 (75%)		3 (25%)	9 (75%)		1 (8.3%)	11 (91.7%)		2 (16.7%)	10 (83.3%)	

Moreover, we compared relative expression of each gene in tumoral samples between clinicopathological-based categories (Table 4). *SOCS1* and *SOCS2* genes were significantly overexpressed in grade 2 samples compared with grade 1 samples (*P* values of 0.004 and 0.04 respectively), but *SOCS3* had the opposite trend (*P* = 0.01). Moreover, expressions of *SOCS1* and *SOCS2* genes were significantly higher in grade 3 samples compared with grade 1 samples (*P* values of 0.007 and 0.05 respectively). No significant difference was found in expressions of other genes between other clinicopathological-based categories.

**Table 4:** Comparison of expression levels of *SOCS* genes in tumoral tissue of breast cancer patients between clinicopathological-based categories (Mean and SD values of (E^CT_HPRT_/E^CT_target gene_) are presented)

	*SOCS1* expression (mean (SD))	*P* value	*SOCS2* expression (mean (SD))	*P* value	*SOCS3* expression (mean (SD))	*P* value	*SOCS5* expression (mean (SD))	*P* value
Age								
< 55 years vs. ≥ 55 years	496.1 (2.6) vs. 592.9 (2.6)	0.88	1.5 (7.9) vs. 10.9 (17.2)	0.43	199.5 (542.6) vs. 84.7 (372.8)	0.4	16.7 (44.9) vs. 1.1 (4.9)	0.33
ER status								
ER (+) vs. ER (−)	675.4 (2.9) vs. 0.6 (0.8)	0.55	1.2 (7.1) vs. 5.1 (7.7)	0.64	192.5 (545.1) vs. 55.1 (127.2)	0.51	537.3 (3.3) vs. 1.5 (1.5)	0.68
PR status								
PR (+) vs. PR (−)	766.1 (3.1) vs. 2.5 (6.9)	0.42	1.4 (7.5) vs. 4 (5.9)	0.53	216.6 (577.2) vs. 41.5 (102.1)	0.08	609.9 (3.6) vs. 1.2 (1.3)	0.58
HER2 status								
HER2 (+) vs. HER2 (−)	20 (25.1) vs. 781.5 (3.1)	0.41	10.3 (17.8) vs. 1.4 (7.6)	0.51	223.3 (763) vs. 160.9 (411.2)	0.71	3.4 (5.6) vs. 626 (3.6)	0.56
Tumor grade								
Grade 1 vs. 2	3.4 (6.2) vs. 13.7 (23.4)	0.004	6.5 (1.6) vs. 13.8 (20.6)	0.04	596 (735) vs. 25.7 (63.9)	0.01	2.8 (7.7) vs. 3.8 (7.1)	0.08
Grade 1 vs. 3	3.4 (6.2) vs. 8.1 (19.2)	0.007	6.5 (1.6) vs. 10.8 (17.6)	0.05	596 (735) vs. 194.5 (659.3)	0.1	2.8 (7.7) vs. 2.3 (3.9)	0.1
Grade 2 vs. 3	13.7 (23.4) vs. 8.1 (19.2)	1	13.8 (20.6) vs. 10.8 (17.6)	1	25.7 (63.9) vs. 194.5 (659.3)	0.5	3.8 (7.1) vs. 2.3 (3.9)	1

### Correlation between relative expressions of genes

We evaluated correlations between expression levels of genes in both tumoral tissues and ANCTs. Significant correlations were found between expression of *SOCS* genes both in ANCTs and in tumoral tissues except for lack of correlation between SOCS2 and SOCS5 in tumoral tissues (Table 5).

**Table 5:** Coefficients of determination (*R* square) values between expression levels of genes in tumoral tissues and ANCTs

		*SOCS5*	*SOCS3*	*SOCS2*
*SOCS1*	Tumor	0.59**	0.65**	0.81**
	ANCT	0.96**	0.85**	0.52**
*SOCS2*	Tumor	0.02	0.5**	
	ANCT	0.69**	0.88**	
*SOCS3*	Tumor	0.43*		
	ANCT	0.95**		

Data show partial correlation as controlled for age

*Correlation is significant at *P* < 0.05 level, **correlation is significant at *P* < 0.01 level

### Partial correlation between expression of genes and patients’ age and tumor grade

We also assessed the correlation between expression of genes and patients’ age and tumor grade after controlling the effects of each variable. After controlling the effects of confounding factors, no significant correlation was found between expression of genes and patients’ age or tumor grade (Table 6).

**Table 6:** Partial correlation between expression of genes and patients’ age and tumor grade

	*SOCS1*		*SOCS2*		*SOCS3*		*SOCS5*	
	*R*	*P* value	*R*	*P* value	*R*	*P* value	*R*	*P* value
Age (controlled for grade)	− 0.02	0.88	− 0.03	0.84	− 0.06	0.66	− 0.007	0.96
Grade (controlled for age)	− 0.36	0.007	− 0.28	0.02	− 0.18	0.1	− 0.25	0.04

### ROC curve analysis

**Fig. 2:**
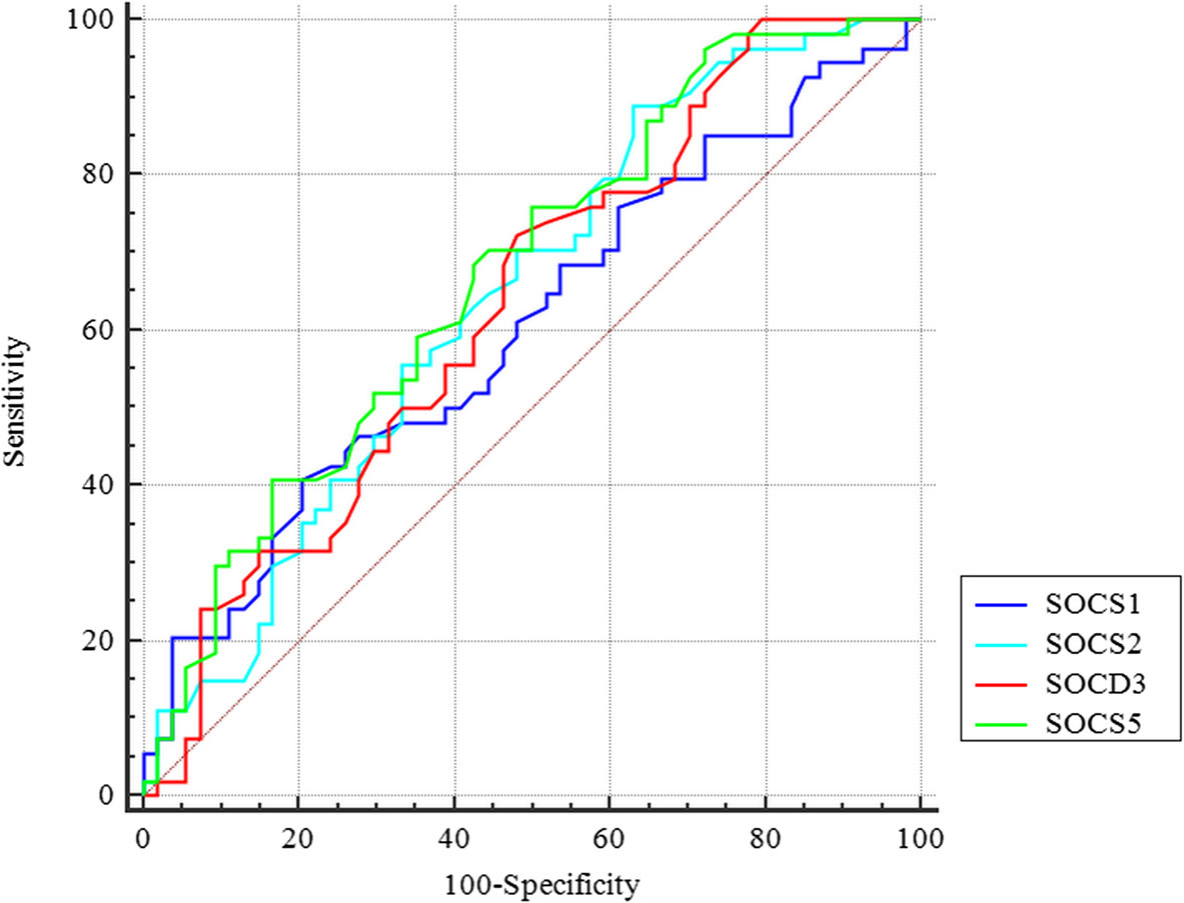
ROC curve for assessment of *SOCS* genes expression levels as diagnostic markers for breast cancer

Based on the ROC curve analysis results, the *SOCS1* and *SOCS2* expression levels had the best specificity and sensitivity values respectively for breast cancer diagnosis (Fig. 2). Combination of transcript levels of all *SOCS* genes improved the AUC value, but such value did not reach the acceptable threshold. Table 7 shows the details of ROC curve analysis.

**Table 7:** The results of ROC curve analysis

	Estimate criterion	AUC	*J* ^a^	Sensitivity	Specificity	*P* value^b^
*SOCS1*	> 2.7	0.6	0.2	40.7	79.6	0.05
*SOCS2*	> − 2.4	0.64	0.25	88.9	37	0.007
*SOCS3*	> − 0.9	0.63	0.24	72.2	51.9	0.01
*SOCS5*	> − 0.8	0.67	0.25	75.9	50	0.0009
Combination of all genes	≤ 0.5	0.68	0.35	64.8	70.4	0.0004

Estimate criterion: optimal cutoff point for gene expression

^a^Youden index,

^b^Significance level *P* (area = 0.5)

## Discussion

SOCS proteins potently regulate the intensity and extent of STAT signals. Absence of SOCS functions would lead to constitutive expression of STAT pathways which in turn triggers cellular transformation, tumor cell invasion, and metastasis [11]. STAT proteins have established role in the development of breast cancer. Downregulation of STAT3 and STAT5a/b has been suggested as a mechanism for anti-proliferative effects of some anti-cancer agents in breast cancer cells [12]. In the present study, we demonstrated significant downregulation of *SOCS1–3* and *SOCS5* genes in breast cancer tissues compared with ANCTs which is in line with the previous findings regarding the role of SOCS proteins in the regulation of STAT proteins and the contribution of STATs in the pathogenesis of breast cancer. Downregulation of *SOCS1* has been demonstrated in numerous malignancies such as prostate cancer, hepatocellular carcinoma, laryngeal carcinoma, multiple myeloma, acute myeloid leukemia, pancreatic cancer, and lymphoma [13]. However, the results of previous studies regarding expression of *SOCS* genes in breast cancer are inconsistent. Raccurt et al. have previously assessed the expression of *SOCS1–3* genes in 17 breast carcinomas, 3 ANCTs, and 10 breast cancer lines and demonstrated higher expression of SOCS1–3 proteins within in situ ductal carcinomas and infiltrating ductal carcinomas compared with normal breast samples. In situ hybridization also confirmed overexpression of *SOCS1–3* transcripts in both tumor tissue and reactive stroma. They suggested that such overexpression might reflect the host/tumor response or be induced secondary to autocrine/paracrine release of growth hormone and prolactin [8]. The inconsistency between our results and Raccurt et al. study can be explained by the low number of samples in their study. As we demonstrated in our study, expressions of *SOCS* genes do not follow a similar pattern in all patients. For instance, while *SOCS1* was downregulated in about two third of breast cancer tissues compared with the corresponding ANCTs, it was upregulated in the remaining samples. This was also true for other *SOCS* genes. Contrary to Raccurt et al., Sasi et al. have assessed expression of *SOCS1–7* transcript levels in 127 breast cancer tissues and 31 ANCTs using real-time PCR and reported no significant difference in their expression between tumoral tissues and ANCTs [7]. Failure to find difference in expression levels of genes between tumoral tissues and ANCTs might be due to dissimilar number of samples in each group. However, they found inverse association between *SOCS1*, *4*, *5*, *6*, and *SOCS7* expressions and TNM stage. Notably, they reported significant associations between higher levels of certain *SOCS* genes and disease-free or overall survival [7].

Our results regarding global downregulation of *SOCS2* in tumoral samples compared with ANCTs are in line with Farabegoli et al. study which demonstrated a role for SOCS2 downregulation in the enhancement of cell proliferation and tumor growth in breast cancer [14]. They also demonstrated positive association between SOCS2 protein expression and low grade, low nuclear grade, and p27 protein [14] which is not consistent with our results. Haffner et al. also demonstrated favorable prognostic value of high SOCS2 expression in primary breast tumors [15].

We hypothesize that *SOCS* downregulation in breast cancer samples as revealed in our study might result in constitutive expression of STAT pathways. Higher expression of STAT genes might contribute to several aspects of tumorigenesis such as cellular transformation, invasion, and metastasis. Alternatively, based on the reported role of SOCS proteins in inhibition of mutant Jak2 and suppression of cytokine-independent signaling [16], downregulation of *SOCS* genes in breast cancer tissues may trigger some cytokine-independent pathways resulting in cell transformation.

Although we did not find any association between expression of *SOCS* genes and TNM stage, we found higher levels of *SOCS1* and *SOCS2* genes in grade 2 and 3 samples compared with grade 1 samples but lower levels of *SOCS3* in grade 1 samples compared with grade 2 samples. Sasi et al. have previously shown downregulation of *SOCS7* expression in higher tumor grades [7]. The observed differences in expression of *SOCS* genes between different pathological grades might reflect specific roles of *SOCS* genes in certain grades of malignancy. One might classify *SOCS* genes to certain groups based on their relative expression in different grades of breast cancer. However, future studies are needed to clarify the practical significance of such observation.

The reported downregulation of *SOCS3* in our study is in line with the Barclay et al. study which demonstrated the antiproliferative role of this gene via inhibition of STAT3 expression and suppression of STAT5 phosphorylation in breast cancer cells [17].

We also demonstrated significant downregulation of *SOCS5* in tumoral tissues compared with ANCTs. Kario et al. have previously shown overexpression of SOCS5 in cells following treatment with epidermal growth factor (EGF). They also reported the effect of SOCS5 on downregulation of epidermal growth factor receptor (EGFR) expression through enhancement of EGFR degradation [18]. Considering the role of EGFR and its downstream pathway in regulation of epithelial-mesenchymal transition, migration, and tumor invasion in breast cancer and the availability of drugs that target this pathway [19], alterations in the expression of *SOCS5* in breast cancer might be involved in the response of patients to such targeted therapies.

The observed downregulation of *SOCS* genes in breast cancer tissues compared with ANCTs might be due to either epigenetic or genetic changes. Sutherland et al. have reported *SOCS1* promoter hypermethylation in 9% of breast cancer samples [5]. On the other hand, deleterious *SOCS1* mutations have been detected in both primary mediastinal B-cell lymphoma and classical Hodgkin lymphoma [20]. Considering the role of growth hormone and prolactin in regulation of SOCS genes expression [8, 17], any change in the secretion of these hormones in the tumor microenvironment might also alter SOCS expression.

We also demonstrated significant correlations between expression of *SOCS* genes both in ANCTs and in tumoral tissues except for lack of correlation between *SOCS2* and *SOCS5* in tumoral tissues which suggest the presence of a similar regulatory mechanism for their expression.

## Conclusion

In spite of significant difference in expression levels of *SOCS* genes between tumoral tissues and ANCTs, none of *SOCS* genes had adequate sensitivity and specificity to be used as a diagnostic biomarker.

Taken together, in spite of frequently reported alterations of *SOCS* genes in human malignancies, the data regarding expression of these genes in breast cancer is inconclusive which necessitates design of further studies with larger sample sizes to elaborate their function in this type of human cancer.

## References

[1] Cooney RN (2002). Suppressors of cytokine signaling (SOCS): inhibitors of the JAK/STAT pathway. Shock.

[2] Rottapel R, Ilangumaran S, Neale C, La Rose J, Ho JM, Nguyen MH, Barber D, Dubreuil P, de Sepulveda P (2002). The tumor suppressor activity of SOCS-1. Oncogene.

[3] Yoshikawa H, Matsubara K, Qian GS, Jackson P, Groopman JD, Manning JE, Harris CC, Herman JG (2001). SOCS-1, a negative regulator of the JAK/STAT pathway, is silenced by methylation in human hepatocellular carcinoma and shows growth-suppression activity. Nat Genet.

[4] Kim Moon-Hong, Kim Moon-Sun, Kim Wonwoo, Kang Mi Ae, Cacalano Nicholas A., Kang Soon-Beom, Shin Young-Joo, Jeong Jae-Hoon (2015). Suppressor of Cytokine Signaling (SOCS) Genes Are Silenced by DNA Hypermethylation and Histone Deacetylation and Regulate Response to Radiotherapy in Cervical Cancer Cells. PLOS ONE.

[5] Sutherland Kate D, Lindeman Geoffrey J, Choong David Y H, Wittlin Sergio, Brentzell Luci, Phillips Wayne, Campbell Ian G, Visvader Jane E (2004). Differential hypermethylation of SOCS genes in ovarian and breast carcinomas. Oncogene.

[6] Evans M K, Yu C-R, Lohani A, Mahdi R M, Liu X, Trzeciak A R, Egwuagu C E (2006). Expression of SOCS1 and SOCS3 genes is differentially regulated in breast cancer cells in response to proinflammatory cytokine and growth factor signals. Oncogene.

[7] Sasi W, Jiang WG, Sharma A, Mokbel K. Higher expression levels of SOCS 1,3,4,7 are associated with earlier tumour stage and better clinical outcome in human breast cancer. BMC cancer. 2010;10:178. 10.1186/1471-2407-10-178 PMC2876081 20433750

[8] Raccurt M, Tam S P, Lau P, Mertani H C, Lambert A, Garcia-Caballero T, Li H, Brown R J, McGuckin M A, Morel G, Waters M J (2003). Suppressor of cytokine signalling gene expression is elevated in breast carcinoma. British Journal of Cancer.

[9] Smolkova Bozena, Mego Michal, Horvathova Kajabova Viera, Cierna Zuzana, Danihel Ludovit, Sedlackova Tatiana, Minarik Gabriel, Zmetakova Iveta, Krivulcik Tomas, Gronesova Paulina, Karaba Marian, Benca Juraj, Pindak Daniel, Mardiak Jozef, Reuben James M., Fridrichova Ivana (2016). Expression of SOCS1 and CXCL12 Proteins in Primary Breast Cancer Are Associated with Presence of Circulating Tumor Cells in Peripheral Blood. Translational Oncology.

[10] Jiang M, Zhang WW, Liu P, Yu W, Liu T, Yu J. Dysregulation of SOCS-Mediated Negative Feedback of Cytokine Signaling in Carcinogenesis and Its Significance in Cancer Treatment. Frontiers in immunology 2017;8:70. 10.3389/fimmu.2017.00070 PMC5296614 28228755

[11] Darnell JE (2005). Validating Stat3 in cancer therapy. Nat Med.

[12] Lim EJ, Hong DY, Park JH, Joung YH, Darvin P, Kim SY, Na YM, Hwang TS, Ye SK, Moon ES (2012). Methylsulfonylmethane suppresses breast cancer growth by down-regulating STAT3 and STAT5b pathways. PLoS One.

[13] Inagaki-Ohara K, Kondo T, Ito M, Yoshimura A (2013). SOCS, inflammation, and cancer. JAKSTAT.

[14] Farabegoli F, Ceccarelli C, Santini D, Taffurelli M (2005). Suppressor of cytokine signalling 2 (SOCS-2) expression in breast carcinoma. J Clin Pathol.

[15] Haffner MC, Petridou B, Peyrat JP, Revillion F, Muller-Holzner E, Daxenbichler G, Marth C, Doppler W (2007). Favorable prognostic value of SOCS2 and IGF-I in breast cancer. BMC Cancer.

[16] Haan S, Wuller S, Kaczor J, Rolvering C, Nocker T, Behrmann I, Haan C (2009). SOCS-mediated downregulation of mutant Jak2 (V617F, T875N and K539L) counteracts cytokine-independent signaling. Oncogene.

[17] Barclay JL, Anderson ST, Waters MJ, Curlewis JD (2009). SOCS3 as a tumor suppressor in breast cancer cells, and its regulation by PRL. Int J Cancer.

[18] Kario E, Marmor MD, Adamsky K, Citri A, Amit I, Amariglio N, Rechavi G, Yarden Y (2005). Suppressors of cytokine signaling 4 and 5 regulate epidermal growth factor receptor signaling. J Biol Chem.

[19] Masuda H, Zhang D, Bartholomeusz C, Doihara H, Hortobagyi GN, Ueno NT (2012). Role of epidermal growth factor receptor in breast cancer. Breast Cancer Res Treat.

[20] Weniger MA, Melzner I, Menz CK, Wegener S, Bucur AJ, Dorsch K, Mattfeldt T, Barth TF, Moller P (2006). Mutations of the tumor suppressor gene SOCS-1 in classical Hodgkin lymphoma are frequent and associated with nuclear phospho-STAT5 accumulation. Oncogene.

